# Implementation of a food retail intervention to reduce purchase of unhealthy food and beverages in remote Australia: mixed-method evaluation using the consolidated framework for implementation research

**DOI:** 10.1186/s12966-022-01377-y

**Published:** 2023-02-17

**Authors:** Julie Brimblecombe, Bethany Miles, Emma Chappell, Khia De Silva, Megan Ferguson, Catherine Mah, Eddie Miles, Anthony Gunther, Thomas Wycherley, Anna Peeters, Leia Minaker, Emma McMahon

**Affiliations:** 1 Department of Nutrition Dietetics and Food, Monash University, Level 1 264 Ferntree Gully Rd, Notting Hill, Victoria, 3168 Australia; 2 Menzies School of Health Research, Charles Darwin University, Royal Darwin Hospital Campus, Building 58 Rocklands Drive, Tiwi, NT 0810 Australia; 3 School of Public Health, Faculty of Medicine, The University of Queensland, Public Health Building, Level 4, Herston, QLD 4006 Australia; 4 Arnhem Land Progress Aboriginal Corporation, 70 O’Sullivan Cct, East Arm, NT 0828 Australia; 5 Dalhousie University, 5850 College Street, Second Floor, PO Box 15000, Halifax, NS B3H 4R2 Canada; 6 University of Toronto, 155 College Street, Toronto, ON M5T 3M7 Canada; 7 Alliance for Research in Exercise, Nutrition and Activity (ARENA), University of South Australia, North Terrace, Adelaide, South Australia 5000 Australia; 8 Institute for Health Transformation, Deakin University, Geelong, VIC Australia; 9 School of Planning, University of Waterloo, 200 University Avenue West, Waterloo, ON N2L 3G1 Canada

**Keywords:** Implementation science, Food retail, Food environment, Remote stores, CFIR, Nutrition, Indigenous health, Mixed method

## Abstract

**Background:**

Adoption of health-enabling food retail interventions in food retail will require effective implementation strategies. To inform this, we applied an implementation framework to a novel real-world food retail intervention, the Healthy Stores 2020 strategy, to identify factors salient to intervention implementation from the perspective of the food retailer.

**Methods:**

A convergent mixed-method design was used and data were interpreted using the Consolidated Framework for Implementation Research (CFIR). The study was conducted alongside a randomised controlled trial in partnership with the Arnhem Land Progress Aboriginal Corporation (ALPA). Adherence data were collected for the 20 consenting Healthy Stores 2020 study stores (ten intervention /ten control) in 19 communities in remote Northern Australia using photographic material and an adherence checklist. Retailer implementation experience data were collected through interviews with the primary Store Manager for each of the ten intervention stores at baseline, mid- and end-strategy. Deductive thematic analysis of interview data was conducted and informed by the CFIR. Intervention adherence scores derived for each store assisted interview data interpretation.

**Results:**

Healthy Stores 2020 strategy was, for the most part, adhered to. Analysis of the 30 interviews revealed that implementation climate of the ALPA organisation, its readiness for implementation including a strong sense of social purpose, and the networks and communication between the Store Managers and other parts of ALPA, were CFIR inner and outer domains most frequently referred to as positive to strategy implementation. Store Managers were a ‘make-or-break’ touchstone of implementation success. The co-designed intervention and strategy characteristics and its perceived cost–benefit, combined with the inner and outer setting factors, galvanised the individual characteristics of Store Managers (e.g., optimism, adaptability and retail competency) to champion implementation. Where there was less perceived cost–benefit, Store Managers seemed less enthusiastic for the strategy.

**Conclusions:**

Factors critical to implementation (a strong sense of social purpose; structures and processes within and external to the food retail organisation and their alignment with intervention characteristics (low complexity, cost advantage); and Store Manager characteristics) can inform the design of implementation strategies for the adoption of this health-enabling food retail initiative in the remote setting. This research can help inform a shift in research focus to identify, develop and test implementation strategies for the wide adoption of health-enabling food retail initiatives into practice.

Trial Registration.

Australian New Zealand Clinical Trials Registry ACTRN 12,618,001,588,280.

**Supplementary Information:**

The online version contains supplementary material available at 10.1186/s12966-022-01377-y.

Contributions to the literature

- There is a paucity of evidence on what implementation strategies can enhance the uptake of empirical evidence into food retail practice for public health gain.
- We found through evaluation of a multi-store food retail intervention randomised controlled trial that implementation was influenced by multiple contextual factors internal and external to the food retail organisation, the individual characteristics of food retailers, the processes in place to engage and support the food retailers, and the co-designed fit-for-context intervention.
- These findings contribute to recognised gaps in the literature, including what implementation strategies may support effective adoption of food retail public health interventions and how these may differ for different contexts.
- Readiness to adopt health-enabling food retail interventions by retail businesses, in a context of limited or no public policy to restrict the promotion of unhealthy food, may more readily occur where there is a strong sense of social purpose.

## Background

Dietary factors, such as low fruit, vegetable and fibre intake, and high sodium and sugar sweetened beverage intake, are leading modifiable contributors to the global burden of disease [1]. Food retail settings, including supermarkets, grocery stores and corner stores, provide a significant proportion of the food available to households and are a major influence on population diet [2]. How food is made available, placed and promoted in-store strongly influences consumer buying decisions [2–9]. In the last decade, there has been an increased public health effort to modify food retail practice to promote more health-enabling options to customers [10–16].

Multicomponent interventions using behaviour change communication techniques in combination with modification of the store architecture (e.g., manipulation of where food and beverage products are placed in the store) have been popular intervention approaches [17–19]. Although evidence on the effectiveness of these approaches is rapidly growing, there is a paucity of information about the implementation strategies needed for the translation of this evidence into practice [18, 20–24]. Research to date has focused on process evaluation (evaluating dose, reach and fidelity of interventions and barriers and enablers to implementation) and on how to motivate and work with retailers to achieve improved nutritional outcomes [20–25]. However, it is critical to know from the perspective of business operators (the retailer in the instance of this study) how promising interventions might be practically implemented in order to optimise both favourable business and public health outcomes [18, 26–28]. This evidence can then advance knowledge on what implementation strategies may be effective for the successful implementation of health-enabling food retail interventions, which at present is wanting in food retail intervention research [24].

A number of reviews have identified the array of factors influencing the implementation of health-enabling food retail interventions [18, 24, 29]. Houghtaling et al. (2019) conducted a review of 22 US studies that examined the perspective of food retailers on in-store interventions to improve nutrition outcomes [18]. They used the socio-ecological framework to identify factors influencing retailer decision-making at different levels (individual, interpersonal, environmental, community, systems or sectors, policy, and norms/values). Factors identified included retailers’ knowledge and skills, retailer-customer relationship, retailer preference for promotional type activities, perceived consumer demand and taste preferences, product availability, supplier arrangements, time barriers, employee turnover, business risk concerns, government policies, retail management factors and community needs.

Middel et al. (2019) [29] applied a systems innovation and the consolidated framework for implementation research (CFIR) to 41 articles, to identify barriers and facilitators to nutrition intervention in food retail stores and identified a similar array of factors to those reported by Houghtaling including consumers’ preferences and demands, relationships, supplier issues, business risk concerns, retailer knowledge, and time barriers. A recent review of 25 reviews by Gupta et al. (2022) [24] investigated factors influencing implementation of healthy food retail interventions, and also found that retailers’ and consumers’ knowledge and food preferences, relationships, profitability, store infrastructure, organisational support including resources, and enabling policies were influencing factors.

Whilst these studies have built much needed evidence on the array of factors influencing implementation, there has been little focus on how these factors can inform implementation strategies to support the effective and sustained implementation of health-enabling food retail interventions [24]. To advance knowledge and theory building on implementation effectiveness [29, 30], tools that offer an overarching typology can guide researchers to identify variables most salient to implementation effectiveness from a public health and retailer perspective [30]. The CFIR offers such a framework [30, 31]. It comprises 39 constructs common to existing theories on successful implementation, organised into five major domains and allows for factors at multiple levels of influence to be explored: intervention characteristics (i.e., the attributes of the intervention that influence success of implementation), outer setting (i.e., the external influences on implementation), inner setting (i.e., characteristics of the implementing organisation), characteristics of the individuals involved that may affect implementation, and the process of implementation [30].

We applied the CFIR to a novel retail food environment intervention, the Healthy Stores 2020 study [32, 33], to identify variables salient to implementation. The Healthy Stores 2020 study was a pragmatic randomised controlled trial (RCT) that aimed to restrict merchandising (i.e., the display, promotion and stocking) of targeted unhealthy products while continuing merchandising of healthy products in stores in very remote regions of Australia [32, 33]. It was a joint initiative involving researchers and a multi-store food retail organisation, the Arnhem Land Progress Aboriginal Corporation (ALPA) [34]. The 12-week intervention was co-designed with the input of retailers, academics and store owners, managers and staff and involved a 7-point strategy that limited merchandising of targeted unhealthy food and beverages [33]. The trial was successfully implemented and resulted in a statistically significant 2.8% reduction in percent free sugars to energy in food and beverages sold (primary outcome) and a 12.6% reduction in sales of sugar sweetened beverages [33].

To inform translation of the Healthy Stores 2020 innovation to practice and policy, this paper reports on our assessment of factors influencing implementation of the Healthy Stores 2020 strategy from the perspective of retailers. Through its application of a standardised implementation assessment framework, this study can help build global evidence on the implementation strategies needed to optimise the adoption of retail food environment intervention evidence in to practice for public health gain.

## Method

### Aim and design

This study was conducted alongside a pragmatic RCT aimed to test the effectiveness of the Healthy Stores 2020 strategy in achieving a reduction in free sugars to energy that is reported elsewhere [33]. For the purpose of the study reported herein, a convergent mixed method design [35] was used to identify factors influencing implementation, informed by data on i) adherence to the Healthy Stores 2020 strategy, and ii) retailer implementation experience; and interpreted through the lens of the CFIR and the insider knowledge of the research team. The research team have extensive experience in public health food retail with the majority having specific experience in the context of remote Australia. The CFIR guided analysis, interpretation and reporting.

#### Theoretical framework

We approached this study from a social constructivist position [36] where we sought to understand factors influencing implementation from the lived experience of the retailers (Store Managers) as informed by the CFIR constructs and within the context of remote stores in the Northern Territory and Northern Queensland, Australia. We were equally interested in factors common to these experiences across the different stores that could inform evidence translation. Adherence to the intervention strategies was viewed as an objective and measurable construct; and implementation overall was seen as a socially constructed concept dependent on context, the retailer experience of implementation, and our interpretation of this with our insider knowledge.

### Study setting

This study was conducted in partnership with ALPA, a private sector not-for-profit business and Australia’s largest Indigenous corporation [34]. ALPA headquarters is in Darwin, Northern Territory (NT). At the time of the design stage of this study (2017), ALPA owned or managed 25 stores in 24 communities across the NT and Northern Queensland. The six stores owned by ALPA were in five NT communities. Each is represented by a community elected ALPA board director and a traditional land owner; who make up the all-Aboriginal ALPA board. ALPA also owned six Island and Cape stores in Queensland (QLD), governed by the Island and Cape board. Additionally, ALPA had management agreements with 12 stores in NT communities and with one in QLD. These ALPA managed stores are owned by Aboriginal corporations and directed by local boards comprising local community members (Store boards). Participating communities ranged in size from 220 to 2560 people with a majority (> 85%) of Aboriginal and/or Torres Strait Islander Peoples [37]. These communities are located in areas of Australia classified as very remote due to restricted services because of geographical distance from urban centres [38], although one community in QLD was in an outer regional area. For many of the ALPA store staff and customers, English is spoken as a third or fourth language. Fifteen of the communities with ALPA owned or operated stores have competitor retail outlets within or in close distance to the community. Stock is distributed weekly to communities by either sea, air or road.

#### Store governance

Store Boards in the ALPA owned and ALPA managed stores set policies and procedures for their stores. Store Managers of ALPA owned and/or managed stores (whom are mostly non-Indigenous and not from the communities) are employed by ALPA and governed by ALPA policies and procedures. Managers are responsible for stock ordering and management, store financial management and housekeeping, employment, training and management of staff (most of whom are Indigenous), and upholding ALPA’s values including nutrition, safety, training and fair employment. ALPA stores are grouped by geographical regions with an Area Manager responsible for each group. Area Managers make regular store visits to report to Store Boards and support Store Managers and store teams, review operations and ensure standards and compliance. At the time of the study, ALPA employed one Nutritionist (Health and Nutrition Manager) with responsibility for the development, implementation and evaluation of the ALPA Health and Nutrition Policy. ALPA’s supply contracts with food manufacturers and wholesalers are managed by a Merchandising Manager. At the time of this study, ALPA had a service contract with a beverage company who for the purpose of the study was requested by ALPA to provide planograms (a visual of stock and facing layout) for the drink refrigerators according to the study strategy.

#### Study context

The partnership with ALPA for this study stemmed from a long, over two-decade relationship between a number of the research team members and ALPA. ALPA has a strong social purpose and developed a Health and Nutrition strategy in the 1980s for its stores and has since subsidised the freight cost on fruit and vegetables and supported nutrition promotion activities in their stores and the communities they serve. ALPA’s Health and Nutrition strategy has objectives to increase the availability and affordability of nutritious foods and increase understanding of health, good food, and nutrition of the community. Prior to Healthy Stores 2020, ALPA had considered a policy to not stock unhealthy products at end-of-aisle and front-of-store. Sugar reduction was seen as a priority by the ALPA Board and by community representatives, many who had voiced concern to members of the research team that high sugar intakes are contributing to adverse health outcomes in their communities. A previous trial conducted by the research team in 2015 with ALPA (the SHOP@RIC trial) [39] found that a 20% price discount on fruit, vegetables, bottled water and artificially-sweetened soft drinks increased sales of these products and resulted in increases in food purchased overall including some unhealthy foods. At a stakeholder workshop to present and discuss the findings of this study, ALPA expressed their interest in testing strategies to reduce sales of unhealthy foods. The ALPA end-of-aisle and front-of-store policy therefore provided a starting point for the Healthy Stores 2020 strategy design.

#### Recruitment of stores for Healthy Stores 2020.

All 25 stores owned and/or managed by ALPA at the time of the study were invited to participate. Twenty stores across 19 communities consented to participate and were then randomised to intervention and control groups, as previously described [32].

### Participant characteristics

Participants for the study described herein were the 20 consenting stores (for adherence data) and each primary Store Manager for each of the ten intervention stores at the time of data collection (for retailer experience data).

### Intervention strategy

The Healthy Stores 2020 intervention with a 7-point strategy (complete strategy) was designed to reduce merchandising of all discretionary food and beverage products and the biggest contributors to free sugars (table sugar, sugar-sweetened beverages, confectionery and sweet biscuits) as detailed in Table 1 and in published supplementary material [40]. Due to ALPA’s concerns about a potential adverse impact on business outcomes, stores with food retail competition within or in close proximity to the community (one in the NT; three in QLD), implemented a modified 6-point strategy (i.e., removal of soft drink units > 600 ml from refrigerators was not implemented).

**Table 1:** Healthy Stores 2020 strategy components

Target	HS2020 Strategy components
**All RED**^**a**^** products**	**1. No promotional activity on RED products** (whilst allowing for promotional activity on healthier products)
	**2. No misleading promotional activity**
	**3. No visible availability of RED products at high traffic areas** (Substitute healthier products)
**Targeted RED products**	**4. Reduced facings table sugar, confectionery & sweet biscuits **(Substitute healthier products)
	**5. Reduced refrigerator space for targeted drinks **(Substitute healthier drinks)
	**6. No RED soft drinks > 600 ml in refrigerators**
	**7. Floor stickers (showing amount of sugar per 1.25L soft drink; promoting water as the healthiest choice) & shelf stripping (giving a warning of high sugar) applied on table sugar, sweet biscuit and confectionery shelves**

^**a**^Products not to be displayed in high traffic areas or promoted. Product classification was informed by ALPA’s Nutrition Policy and the Healthy Stores 2020 Food Reference Guide (published supplementary material[41]), which draws from other resources such as the NT Schools Nutrition Policy drinks classification framework

### Implementation

ALPA were responsible for the implementation of the Healthy Stores 2020 strategy in their stores. A visiting team of two to three people that comprised an ALPA staffer/s and/or a research team member set-up the intervention in each of the ten intervention stores with the input of the Store Managers and the assistance of staff, and Store Boards in some stores. Store Managers and their staff maintained the strategy for 12–13 weeks with support from ALPA staff (i.e., the Nutritionist and Area Manager). Intervention set-up (which took less than half-a-day) was guided by a pre-determined list of modifications determined from an assessment of photographs taken of each store’s layout and product placement at baseline against strategy requirements. The beverage supplier provided pictorial planograms as requested by ALPA to aid compliance with refrigerated beverage layouts, and also allowed reimbursed write-off costs of expired artificially sweetened drinks as part of the pre-existing agreement with ALPA. Each Store Manager was provided with a reference guide developed by ALPA with the research team on which products were permitted to be promoted at intervention set-up [41]. Visiting ALPA staff were required by ALPA to check and rectify non-adherence and communicate this to the ALPA Nutritionist who then rectified this with the respective Store Manager. A researcher made fortnightly phone-calls to Store Managers to collect data on adherence and help rectify instances of non-adherence identified. These processes were designed by ALPA with the research team to support optimal implementation. Control stores continued usual retail practice.

### Data collection

Adherence to each strategy component was assessed using an adherence checklist applied fortnightly by a research team member (EC) via fortnightly phone-interview with Store Managers at a time of their convenience to check adherence (Additional file Table A1) with the first call made within one to three weeks of set-up. Photographic material of relevant store areas, for both intervention and control stores, were also collected by the research team at baseline (T1) and in the last week of the 12-week strategy (T3), and by Store Managers at the time of the fortnightly adherence checks (Additional file Table A1).

Retailer experience was assessed using semi-structured interviews with Store Managers of intervention stores. Researcher (EC) applied the interview guide (Additional file Table A2) within one to three weeks of set-up (T2), midway (T-mid), and in the last week of the 12-week strategy (T3) after completing the adherence checklist with Store Managers. The interview guide comprised 16 questions that asked about perceived impact of the intervention on sales, benefit to store and community, implementation fidelity, intervention characteristics (i.e., components working well, not working well), enablers and barriers to implementation, anticipated barriers, views on continuation of the strategy post-trial, customer response and ideas for future strategies. Responses were written verbatim into a word document and transcripts then uploaded to NVivo 12.64 [42] for analysis. Phone rather than face-to-face interviews were considered appropriate due to: the established rapport between the research team and ALPA, ALPA leadership communicating their support for phone interviews to Store Managers, success with phone interviews by the research team in previous remote store food retail research, and the prohibitive cost of travel to very remote communities in Australia (e.g., a return airfare to a community for example can cost AUD $1200 (at the time of the study)).

### Data analysis

Adherence was summarised for each strategy and a total score calculated (i.e., one point for each strategy component if full adherence is observed at all time-points with a total possible score of 6 (for modified 6-point strategy) or 7 (complete 7-point strategy), converted to a percentage). Baseline (T1), following set-up (T2) and end of strategy (T3) changes in shelf space (facings) of targeted products were calculated from photographic data. This involved the counting of facings for each of the targeted products by the research team using photographic data. Criteria for calculating confectionery, sweet biscuit and drink facings were developed as detailed in Additional file Table A3. Drinks were classified into ‘green/amber’ or ‘red’. Targeted beverages were ‘red’ beverages. Each drink unit visible at the front of the fridge was considered as one ‘facing’. Multipacks of water were counted according to the number of bottles facing the front of the fridge. Duplicate products stacked on top of each other, as was sometimes in the case of juice boxes, were counted as single facings. The number of fridge doors were counted for each store at each time point using the photographic data. If a whole fridge (or entire fridge door) compared to other time points appeared to be missing from the photographic data, data were imputed with data from the next closest timepoint available (including the fortnightly photos taken by the Store Manager where provided). Data checks on drinks were completed by a second researcher on six stores for T1, T2 and T3 and on confectionery and sweet biscuit data for five stores. Where there was a difference of > 3%, both data sets and the accompanying photos were rechecked. Adherence outcomes were compared and contrasted with interview data where relevant to assist with interpretation.

Deductive thematic analysis of interview data was conducted using a framework approach [43]. First, familiarisation with interview transcripts occurred, where EC and JB conducted multiple reviews of the data. Second, indexing was completed by BM using CFIR codes with two additional codes to capture ‘suggested strategies’ and ‘customer response’. A codebook with CFIR construct definitions adapted to suit the data set (as shown in Table 2) was developed by EC, BM and JB. NVivo was used to assign references in the transcripts to codes according to the codebook [42]. The first three transcripts were independently coded by BM and JB. Both authors discussed the coding to ensure consistent application of the codebook by BM, who then coded all transcripts. Next, each coded reference was annotated to summarise its essence. Annotations were then copied into Microsoft Excel, organised by timepoint and community, with a worksheet created for each code.

**Table 2:** Summary of interview data by CFIR domain and construct, adapted construct name, and summarised finding with illustrative quote

Domain	CFIR Constructs	Adapted construct name/data included	Summarised finding	Illustrative quote
**Intervention characteristics**	Intervention Source	Nil	• Intervention perceived as a 'study' • External entity perceived as funder/evaluator	*“I say we’re still undergoing the study, show them the stickers, say there’s not too much I can do at this stage”* (M3, T2)
	Evidence Strength and Quality^#^	N/A	N/A	N/A
	Relative advantage	Included managers’ perception of the sales effect of the intervention	** + Overall sales unaffected**,—Overall sales decrease ** + Reduction in 1.25L sales,** ** + Increase in sales of SF**^a^ **and smaller volume SSB**^b^ + Increased water sales + Confectionery sales reduction when relocated from POS^c^ **-No reduction in biscuit and sugar sales** + Change in sales mix,—No change in sales mix*	*“Don’t believe it’s had an impact [on overall sales]…think the sales mix has changed.”* (M1, T2) *“We’re selling a lot more diet stuff…[previously] 2–3 pallets of Coke to 1 pallet Diet Coke, now it’s the opposite”* (M2, T2)
	Adaptability	Included references to flexibility to not implement 1.25L strategy, and references to adaptability of strategies to store infrastructure	+ 1.25L exclusion option helpful + Fridge layout requirements adaptable - Fridge layout requirements not adaptable	*“We’re not removing the 1.25L – the second supermarket in community that has us in competition, we were worried that if we made a change with our display, they would get our customers, to shop there”* (M7, T2)
	Trialability	Nil	** + Desire to continue HS2020**^**e**^** after 12-week period** + Feasible for set trial period	"*I’d argue that we should continue – it’s not affecting us – we shouldn’t stop any"* (M1, T2)
	Complexity	Included barriers/ challenges regarding specific strategy elements	** + No challenges for store managers or staff**, + **No significant barriers to complete strategy** **- Inability to minimise write-offs** **- Staff difficulty in following layout, + Manager support for staff accustoming to layout changes** - Language/literacy barrier,—Initial planograms late/incorrect + Planograms helpful to follow layout - Stocking shelves more frequently **-** Adjusting stock control - Customer familiarisation to strategy - Staff turnover/absence	*“Hardest thing is sugary product not going on special at the front, not on the counter or whatever. In retail it makes sense to put specials on when they’re near the date. Writing off more stock, takes time, costs money”* (M8, T2)
	Design Quality and Packaging	Included aesthetic changes in the store due to the intervention	+ Overall HS2020^e^ package well presented ** + Store layout changes effective (particularly 1.25L SSB**^b^**)** **- Shelf stripping unnoticed** **-** Floor stickers unnoticed + Floor stickers noticed • Suggested further layout changes	“*The way the project has been put together is really good – we’re still selling everything I’ve just changed the set up”* (M2, T2) *“I honestly can’t say the shelf stripping made any difference towards the end. Whether it’s just part of the furniture… Or we could change floor stickers more regularly, because something different is more noticeable. Otherwise it becomes same same. Different things can be done*” (M2, T2)
	Cost	Nil	** + No additional financial costs,** + Supplier covering cost of expired SF^a^ drinks - Write-offs causing increased costs ** + No extra time cost,** • Initial time cost only - Increased time cost of restocking smaller facings - Initial stock control/supply issues + No stock control/supply issues*	*We are getting more wastage in sugar free drinks; [softdrink supplier] are wearing the cost of that. Other than that, no not really [any extra costs].”* (M1, T2) *“Maybe a bit [time consuming] at the beginning but not in a negative way.”* (M4, T2)
**Outer Setting**	Patient Needs and Resources	Community Needs and Resources	** + Customers need health improvement/sugar reduction** • Customers need education and promotion of healthy choices + Customers need HS2020^e^ - No customer need for HS2020^e^ • Customers need/desire cold drinks, • Customers need freedom of choice	*“I hope it helps with promoting more low sugar, for the community, we have a lot of diabetics, to get kids into the routine of low sugar/no sugar. Or water.”* (M9, T2)
	Cosmopolitanism	Includes references to any external organisations	+ Collaboration with local health services helpful + Collaboration with suppliers helpful - Collaboration with suppliers unhelpful	“*An old man came up to me asked where the no sugar coke was… he also asked where the no-sugar sugar was!…the clinic started this.”* (M6, T2)
	Peer Pressure	Includes references to other stores, or lack of, in community. CFIR code refers to pressure to implement the intervention; our code refers to pressure *not* to do so	+ No competition facilitates success - Competing stores prevent full strategy implementation + Promoting health gives competitive edge over other stores	*“We have two other places we’re competing with. Supermarket and a takeaway in town, they both have Coke in the fridges, and there’s vending machines…out the front of people’s houses. Competition-wise. We’re happy to do 50:50, but taking it [SSB* > *600 ml] out of the fridge, not quite ready for that yet.* (M9, T2)
	External Policies and Incentives	Nil	• Unspecified nutrition policy/regulations referenced as pre-existing before HS2020^e^, limiting promotion of unhealthy products	*“We were told we weren’t allowed junk, we used to have lollies, chocolates at the front counter…we changed this before healthy stores started, when shop was relayed”* (M9, T2)
**Inner Setting**	Structural Characteristics^#^	N/A	N/A	N/A
	Networks and Communications	Nil	** + Remote communications between store manager and internal management/nutritionist helpful** + Site visits by internal nutritionist helpful + Relationship between manager with store board helpful - Inconsistent directives from internal management unhelpful	*“Having [nutritionist] there, working with her, to relay the stock. As well just to have her out to chat, previous to them coming out, they [nutritionist and area manager] both called, been very clear in why, what and how this happened. Made me feel confident to customer about why we’re doing this”* (M2, T2)
	Culture^#^	N/A	N/A	N/A
	Implementation Climate			
	1) Tension for change	Community health/diet/ discretionary food marketing concerns of managers	** + Change needed for community health/high sugar intakes**, + Change needed due to customer demand + Change needed to reinforce public health messages - No tension for change	*“Healthwise [HS2020*^*e*^*] would help them a great deal because they’re not having so much sugar, most of them are diabetic”* (M5, T2)
	2) Compatibility	Relating to specific store type, location and needs of the specific community	** + HS2020**^**e**^** compatible to stores** - HS2020^e^ a poor fit: pre-existing healthy community/store* - HS2020^e^ a poor fit: small store type* - 1.25L SSB strategy a poor fit: competitor store in community*	*“I don’t see there’s any issues having them [HS2020*^*e*^* strategies] in place in any & every store.”* (M10, T2)
	3) Relative Priority	Nil	** + Expected health outcomes outweigh nil store benefit** + Expected health outcomes will bring long term store benefits	*“Sales wise it’s a hindrance. But for the benefit of their health its needed.”* (M4, T2)
	4) Organisational Incentives and Rewards	Included incentives of increased stature or respect	• Respect lost if manager was noncompliant with HS2020^e^ goals	“*Usually discount [confectionery], but couldn’t promote products. Well it would be been frowned upon”* (M6, T2)
	5) Goals and Feedback	Included reference to goals and compliance data being communicated to store managers	** + HS2020**^**e**^** goals clearly communicated to store manager** + Manager communicated goals to store staff + Manager received goal-related feedback regarding performance • Manager desiring more goal-related feedback regarding performance	*“[ALPA nutritionist] has definitely been a big help; in letting us know what was expected beforehand, good communication”* (M7, T2)
	6) Learning Climate	Included comments regarding relationship between store managers and staff, and staff members’ response to HS2020	** + Managers supervises/protects/ teaches staff in new strategies** ** + Staff perceived as an enabler** + Need for staff members’ involvement acknowledged - Need for staff members’ involvement not acknowledged*	*“Keeping up, making them [staff] understand full sugar on shelf; make them read the labels… Walk with them, make sure they follow the strategy”* (M6, T-mid)
	Readiness for Implementation			
	1) Leadership Engagement	Included reference to store managers involvement and commitment to HS2020	** + Manager is committed and involved in HS2020**^**e**^ -Manager uncommitted to HS2020^e^	*“I’m excited to see the results, we wanna try and help people not have so much sugar. The way the project has been put together is really good – we’re still selling everything I’ve just changed the set up”* (M2, T2)
	2) Available Resources	Included comments regarding planograms and other resources	** + Planograms helpful,** -Planograms initially unhelpful + Fridge signage informing customers helpful + HS2020^e^ summary guide helpful with manager turnover	*“Have a read through about how it all worked. It was useful knowing what you guys targeted, what to look for with what’s got sugar”* (M9, T2)
	3) Access to Knowledge and Information	Included comments regarding SSD^d^	** + Adequate access to SSD**^**d**^ - Inadequate access to SSD^d^ to affirm HS2020^e^ effectiveness - Inadequate access to SSD^d^ Initially, to adjust ordering	*“Obviously full sugar, heavy sugar items went down – Coke 1.25, most of the full sugar 1.25 drinks…no sugar and diet definitely went up. and not as much as full sugar went down. Judged this from sales reports from previous months, you can see what’s going on.”* (M6, T2) *“It’s probably a month to catch sales data, work out what’s up what’s down.”* (M2, T2)
**Characteristics of Individuals**	Knowledge and Beliefs about the Intervention	Including opinions on general efficacy of strategy, attitudes and beliefs	** + Belief: HS2020**^**e**^** benefits community** + Belief: HS2020^e^ is easy to implement ** + Belief: HS2020**^**e**^** 1.25L SSB strategy is effective** **- Belief: biscuits/sugar/ signage strategies ineffective** + Belief: HS2020^e^ is overall effective and valuable - Belief: HS2020^e^ is overall ineffective/unnecessary*, • Neutral belief in HS2020^e^ efficacy + Belief: no profit loss to store HS2020^e^ - Belief: profit loss to store with HS2020^e^ + Knowledge: managers familiar with HS2020^e^ principles/facts - Knowledge: managers unfamiliar with HS2020^e^ principles/facts	*“… helps the community consuming less sugar, should help dramatically”* (M1, T2) “*The soft drink sales have certainly gone down. The 1.25. They’re buying the smaller ones. They want something cold.”* (M4, T-mid) “*I don’t think we’ve been successful in reducing the amount of biscuits or sugar, the stripping hasn’t been effective…” (*M7, T-mid)
	Self-efficacy	Included comments regarding managers' confidence in implementing HS2020^e^	** + High confidence in maintaining strategies for study period** ** + High confidence in maintaining strategies post-study period** ** + Managers’ retailing skills are transferrable to HS2020**^**e**^	*“[The strategies are] not any harder than [selling] any other product, just normal retail business. Just no sugar instead"* (M5, T2)
	Individual Stage of Change^#^	N/A	N/A	N/A
	Individual Identification with Organisation^#^	N/A	N/A	N/A
	Other Personal Attributes	Nil	** + Pragmatism** ** + Optimism** ** + Adaptability and resilience** ** + Marketing and retail competency** ** + Knowledgeable in current sales and local purchasing patterns** **-** Not knowledgeable in local language	“O*h absolutely [can continue post-strategy period]. [You mentioned that customers are still asking about the 1.25 s they are just used to it now anyway. They just deal with it, eventually it will run its course, I haven’t given them an end date.”* (M3, T2)
**Process**	Planning^#^	N/A	N/A	N/A
	Engaging			
	1) Opinion Leaders	Nil Combined from CFIR codes: opinion leaders, formally appointed internal implementation leaders, and champions. This refers to leading/influential store managers or store board members	+ Leading managers motivated and supported staff + Leading managers extended strategies post-intervention + Store board motivated store workers	*… just learning where everything goes. And I guess understanding why. We walked around the store with our staff and had a staff meeting. If the manager was engaged we could make sure the staff understood why, if we weren’t engaged it would be been very confusing for the staff as they need to understand why* (M4, T2)
	2) Formally Appointed Internal Implementation Leaders			
	3) Champions			
	4) External Change Agents^#^	N/A	N/A	N/A
	Executing	Adherence	** + All implemented fully** - Short term unintentional noncompliance with drinks layout - Noncompliance after study period	*“All of it is there, stayed the same and now is normal routines.”* (M5, T-mid)
	Reflecting and Evaluating	Included comments regarding team debriefing with their staff, or the value of phone calls to debrief/reflect	+ Phone calls/support from research staff helpful	*“Your [research assistant] calling, your support, calling in and not letting me go…[was most helpful]”* (M6, T-mid)
	Additional code*	Customer Response: Actual responses to the strategies seen or heard by managers	**• No customer response heard** **• Neutral verbal response only** -Negative verbal response + Positive verbal response **• Changed purchasing pattern seen** • No comment/observing of signs/stickers, + Noticed customer looking at signs/stickers	*“Surprisingly we haven’t had much negative response at all, I’m very surprised about that. A few questions about why it’s being done”* (M4, T2)
	Additional code*	Suggested Strategies: Suggestions offered by managers to improve healthy food and drink merchandising in their store	• Improve store floorplan, Improve placement of additional low sugar products • Focus on healthy product promotion • Adaptations of existing merchandising material • Scale up of HS2020^e^ across ALPA, • High-impact campaign to target table sugar • Other	*“Yes but good to know now that it’s [HS2020*^*e*^*] over I can change a couple of things, we’re swapping the nuts and lollies so you get to the nuts first, and the water should be in the first door of the fridge and now it’s the last.”* (M2, T2)

^#^Excluded as no comments relevant (no questions asked about this construct)

**Bold:** Common responses (≥ half of the respondents)

+ Positive feedback

-Negative feedback

• Neutral feedback

^*^Reported by managers not implementing 1.25L SSB strategy

^a^*SF* Sugar free

^b^*SSB* Sugar sweetened beverages

^c^*POS* Point of sale

^d^*SSD* Store sales data

^e^*HS2020* Healthy Stores 2020

**Fig. 1:**
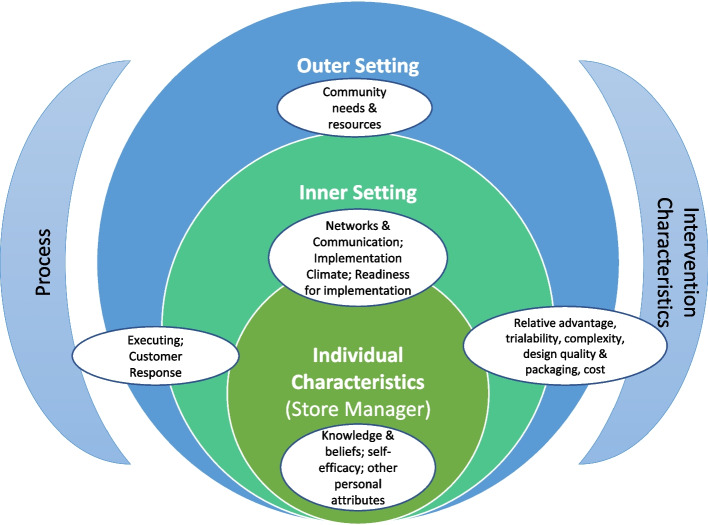
Key factors associated with strategy feasibility across the Consolidated Framework for Implementation Research domains [30] The intervention was seen by most Store Managers as fit-for-purpose to meet the needs of the community (OUTER) and as well packaged to implement (INTERVENTION CHARACTERISTICS). This, together with the strong leadership of ALPA and commitment to the strategy (INNER), good communication and support provided by ALPA during the trial (OUTER and INNER), and less negative customer response than anticipated (PROCESS), enabled Store Managers to use their expertise to integrate the strategy component into their day-to-day work routines once the strategy was set-up (INDIVIDUAL CHARACTERISTICS)

Thematic analysis was then carried out by BM and discussed and cross-checked with JB. This involved looking for consistencies and contrasts across annotations for each code to formulate themes. Themes were added as columns to each of the Microsoft Excel worksheets. Colour highlight was then used to show where a theme directly corresponded with an annotation. Some themes were counted quantitatively, based on their frequency match with an annotation. For other themes, the frequency of annotation-theme match was used to determine the relative importance of the CFIR construct. CFIR constructs with comment from ≥ 50% of respondents, were included in a conceptual diagram (Fig. 1). Convergence of data occurred through an examination by BM and JB of consistencies and inconsistencies between adherence scores and annotations with any inconsistencies noted in the data summaries. Themes for each CFIR code were summarised by BM and JB and reviewed by co-authors (EC, KDS, EMc).

## Results

Adherence checklists and photographic material were collected for all 20 stores. A total of 30 interviews were conducted with some changeover of Store Managers throughout this period. A summary of themes by the CFIR constructs, including constructs with no supporting data, are presented in Table 2. Store Manager quotes are presented using a coding convention of Manager 1–10 and time period T1, T2, T-mid, or T3.

### Adherence

Adherence results are shown in Table 3 and Table A3. Six of the ten stores had an adherence score ≥ 60% using our strict criteria. There was full adherence by all stores to the implementation of stickers/shelf stripping, restricted shelf space for table sugar, and removal of targeted beverages > 600 ml from refrigeration, and near full adherence to no promotions, no misleading promotions, and no availability of unhealthy products in highly visible areas. ‘Complete’ strategy (7-point strategy) stores achieved greater reductions in facings of targeted products than the ‘modified’ strategy (6-point strategy) stores. Facings of targeted products increased slightly on average in control stores. As reported elsewhere [33] and shown in Additional file Table A3, the few occurrences of non-adherence detected for the strategy components — no promotional activity, no misleading promotional activity and no display in high traffic areas — were when one to three product types were detected as promoted (and in most cases these were rectified) and/or misleading drink fridge stripping for example, was placed back in the drink refrigerators by the supplier after it was taken out at set-up.

**Table 3:** Adherence to strategy component for complete and modified strategy and control stores, by store number and percent reduction in facings for confectionery, sweet biscuits and targeted drinks

Strategy component		**Complete 7-point**		**Modified 6-point**^**a**^		**Control**	
		Fully adhered (n of 6)	%	Fully adhered (n of 4)	%	Fully adhered (n of 10)	%
**1. No promotional activity on RED products**		2	NA	2	NA	2	NA
**2. No misleading promotional activity**		3	NA	3	NA	0	NA
**3. No visible availability of RED products at high traffic areas (Substitute healthier products)**		3	NA	3	NA	0	NA
**4. Reduced facings table sugar, confectionery & sweet biscuits (Substitute healthier products)**	Reduced facings of table sugar	6	NA	4	NA	4	NA
	Median % reduction in confectionery facings T1-T3	6	-40	3	-31	3	+ 12
	Median % reduction in in sweet biscuit facings T1-T3	6	-37	2	-12	4	+ 2
**5. Reduced refrigerator space for targeted drinks (Substitute healthier drinks)**	Median percent of refrigerated targeted beverage facings to total refrigerated beverage facings T1/T3	6	60/45	1	56/59	3	58/61
**6. No RED soft drinks > 600 ml in refrigerators**		6	NA	NA	NA	NA	NA
**7. Floor stickers and shelf stripping**		6	NA	4	NA	0	NA

^a^Four stores received the 6-point strategy where RED soft drinks > 600 ml were not removed from refrigeration

### Retailer implementation experiences

#### Intervention characteristics

As shown in Fig. 1, the CFIR constructs of relative advantage (vs alternate strategies), trialability, complexity (ease of implementation), design quality and packaging, and cost (time/financial) were intervention characteristics most associated by Store Managers with strategy implementation success.

Relative Advantage: Most Store Managers kept a close eye on changes in their sales mix and noted change in the mix of beverage sales with little effect of the strategy on overall sales.

*A lot more water walked out the door, water for kids, for themselves. Water out sold coke sales. We actually nearly ran out water* (M9, T2).

*Not having lollies at the registers – lollies, ‘kinder surprises’, now do not sell these at all. Not selling at all. This might impact overall sales – there’s almost no point to order this stuff if it doesn’t sell* (M2, T-mid).

Store Managers of two of the four stores with the 6-point strategy questioned the relative advantage of the Healthy Stores 2020 strategy for their community, and reported to observe no change in their sales mix with the strategy. This was in contrast to the positive effect of the modified intervention on percent sugar to energy shown through the effectiveness trial that we previously reported [33].

*…, people are pretty healthy here anyway, our fridges didn’t need to change as they were already 50% healthy* (M8, T2).

*I’m guessing some of them have worked but we haven’t noticed it* (M9, T-mid).

*Don’t see it having much of an effect at all; we’re a servo so we have a lot of competition: woollies, coles. People will come & buy no sugar coke but we hardly ever sell other no sugar drinks* (M9, T2).

Adaptability, Trialability and Complexity: More than half of the Store Managers reported no implementation challenges and/or barriers and three Store Managers without prompting expressed their desire to continue the Healthy Stores 2020 strategy after the study end. Where challenges with implementation were raised, they related to stock control adjustments, increased frequency of stocking shelves, stock write-offs due to not being able to promote certain unhealthy products even if close to their expiry date, difficulties for some staff in following the new lay-outs (especially for beverages) including for two stores where language barriers impacted the effective communication of the strategy to staff by the Store Managers, high staff turnover, familiarising customers with the changes, and fixed store architecture being not designed in the first place to promote healthier food and beverages.

*Staff are having to restock three times a day instead of once a day* (M1, T2).

*I don’t know whether customers fully understand why [we’ve made the changes], same with the staff. You tell them and they say ‘yes’ but we’re not getting any questions/feedback. The community might be affected by the language barrier* (M2, T2).

Nevertheless, Store Managers consistently affirmed that any challenges experienced with the intervention were not significant enough to prevent the store’s completion of the 12-week strategy and none expressed a desire to stop the strategy. Indeed, one Store Manager commented that the intervention was "Not any harder than [selling] any other product, just normal retail business. Just no sugar instead" (M5, T2).

*Once we communicated what we were doing and what the goal was, the staff found it easy to be on board* (M7, T-mid).

Design quality and packaging: Design elements mentioned by several Store Managers were that the strategy was well presented overall and in a way that benefited both stores and customers. They offered their thoughts on the most effective strategy components and suggestions on how to make strategy components they perceived as less effective, more effective (i.e., change unnoticed shelf stripping and floor stickers to be more noticeable).

*The less confec[tionery] behind the counter might be reducing what the kids are seeing as a final grab before they leave the store. Confec[tionery] is still within arm’s reach of the till. In an ideal world confec[tionery] would be at the back; veg[etables] would be at the front* (M10, T2).

*I don’t think we’ve been successful in reducing the amount of biscuits or sugar, the stripping hasn’t been effective, it would take a pretty high impact education campaign to get people to stop buying kg & kg’s of sugar* (M7, T-mid).

##### Cost

At study completion, half of the Store Managers reported no additional cost to their business as a result of the strategy. Three Store Managers attributed this to the beverage manufacturer crediting expired sugar free drinks back to the store. However, three other Store Managers cited additional product ‘write-offs’ caused increased costs. One Store Manager however stated: “I think if you updated and made sure your stock controls were amended it [write-offs] shouldn’t have been an issue.” (M4, T2).

*Initially getting our ordering levels right, we really had no idea of the impact.. it took us 4–5 weeks before we got our stock levels right. First 3–4 weeks we had a lot of out-of-stocks, in big sellers like 600 mL coke, no sugar [varieties], 250 mL cans. We just weren’t expecting such a big swing around from 1.25 to smaller. First 3–4 weeks every week we’d run out of stock initially.. at least 4 days of the week affected by missing products. Our sales doubled or tripled in some lines. We’re over-ordering now, we deliberately increased order to where we wouldn’t run out. Now I have good sales data I can trim it back to where it should be* (M1, T2).

When asked directly about time costs, half of the Store Managers reported no or negligible extra staff time cost, or were unsure, whilst the other half reported an initial time cost, due to staff training, planning, adjusting orders and set-up, although this was seen by one Store Manager as “not in a negative way” (M4, T2) and as a worthy investment.

*Sugar & confec[tionery] sections are smaller; [and we’re] running out of stock more often.*

#### Outer setting

Community Needs and Resources, as shown in Fig. 1, were the outer setting CFIR construct most frequently referred to by Store Managers in relation to implementation of the strategy.

Community Needs and Resources: Store Managers described a sense of social responsibility for acting on behalf of the community. They were aware of the high burden of chronic disease experienced among their customers, and the potential benefit of the strategy in the prevention and/or management of chronic disease.

*I hope it helps with promoting more low sugar, for the community, we have a lot of diabetics, to get kids into the routine of low sugar/no sugar. Or water* (M9, T2).

They felt there was a need in addition to the strategy for in-store promotion and education on healthy/unhealthy products and to provide customers healthier options within product ranges, with an emphasis on retaining choice. In contrast, one Store Manager who felt the strategy was “a good strategy” believed the community they served did not need such a strategy as they had good health and were already meeting the intervention target of “50% healthy drinks” prior to Healthy Stores 2020 (M8, T2). This store had 52% healthy drinks facings at baseline, meaning they were close to the strategy target of 60% healthy drinks (Additional Table A3).

Cosmopolitanism: Networks with external organisations, including suppliers and local health services, influenced retailer experience. Store Managers described positive and negative dealings with suppliers. For example, one store received planograms provided by the beverage supplier that didn’t align with the store lay-out. This led to a short time of nonadherence with the strategy and frustration of the Store Manager. Another reported that a supplier contract meant that sugar sweetened beverage refrigerators were close to the counter and thereby limited the study’s implementation of no unhealthy food/drinks in high traffic areas (M10, T2).

*[Beverage] planograms were a huge help, helped Yolngu staff focus on what went on which shelf, without that visual it would have been a nightmare…Planograms on fridge doors. They’re essential at any other store. Prior to strategy we weren’t using one, staff took to the new one well* (M1, T2).

Peer Pressure and External Policies and Incentives: Few comments were made that related to these constructs. Two Store Managers commented that retail competition prevented implementation of the complete strategy (i.e., the 7-point strategy) due to concerns of losing sales, while another saw a competitive advantage in having a health-promoting store. Store Managers were aware of ALPA’s pre-existing Health and Nutrition strategy.

*We have two other places we’re competing with…Competition-wise. We’re happy to do 50:50, but taking it out of the fridge; we’re not quite ready for that yet* (M9, T2).

*…hopefully they [customers] will see our healthiness and do their shopping here instead of the other shop* (M7, T-mid).

#### Inner setting

Networks and Communications, Implementation Climate and Readiness for Implementation were the CFIR constructs most frequently referred to by the Store Managers as facilitating implementation.

Networks and Communications: Communication referred to as helpful was that delivered by the Nutritionist, Area Manager and/or research team. Upskilling by the ALPA Nutritionist gave clarity on study expectations and rationale and made Store Managers feel confident with the strategy. Support from ALPA management staff was also seen as useful “to pull sales figures every fortnight” (M2, T2), to “set the stock control” (M10, T2), and to provide handovers to new Store Managers. One Store Manager mentioned the relationship they had with their Store Board as helpful in maintaining the strategy (M2, T2).

*Having [ALPA Nutritionist] there, working with her, to relay the stock. As well just to have her out to chat. Previous to them coming out, they [ALPA Nutritionist] & [Area Manager] both called, been very clear in why what and how this happened. Made me feel confident to communicate to customers about why we’re doing this* (M2, T2).

*There’s a 2020 pack here [merchandising strategy summary guide]. And when you ring up for calls like this time, you ask is this there, and we talk through bits & pieces* (M2, T2).

*Store board members – involved in staff meeting – a board member spoke in language, I’m unsure of translation of what was actually said. [As a result], Staff have been really supportive, they do understand to an extent what’s happening* (M2, T2).

Implementation Climate – Tension for Change, and Compatibility: Most Store Managers commented on strategy alignment with community need and its compatibility with their existing workflow and store systems. One Store Manager stated “I don’t see there’s any issues having them [the strategy] in place in any and every store.” (M10, T2). Two Store Managers however did not agree with this alignment and compatibility. One due to their store already performing well and serving an already “pretty healthy” community (M8, T2), and the other due to operating as “more of a servo [service station]” and having close retail competition (M9, T2).

*We only have a 3-door fridge. It [1.25L drinks] need to be available cold in the cafe, for me to run the café* (M8, T2).

Implementation climate – Relative Priority, and Organisational Incentives and Rewards: Most Store Managers believed that the potential health outcome from the strategy would outweigh potential profit losses and be a win–win for community health and for store business.

*Strategy worked really well…I must admit it was easier for me as I actually believed in this study…if the manager was engaged we could make sure the staff understood why, if we weren’t engaged it would be been very confusing for the staff as they need to understand why* (M4, T2).

Implementation climate – Goals and Feedback, and Learning Climate: Store Managers expected clear communication from ALPA on what was expected of them to achieve strategy adherence. They were motivated by goal-related feedback on their performance and fear of loss of respect if not compliant. In turn, Store Managers took it on to train their staff and identified champions among their staff to help those who struggled with the changed lay-out of products.

*Not everyone is confused, some staff do know what they’re doing so we do get them to help* (M2, T-mid).

Readiness for Implementation – Leadership Engagement, Available Resources, and Access to Knowledge and Information: A number of resources and access to knowledge and information supported Store Managers with the strategy including the supplier planograms, a strategy guide for Store Managers provided by the research team with ALPA, and access to timely sales data to optimise stock control.

*Have a read through about how it all worked. It was useful knowing what you guys targeted, what to look for with what’s got sugar* (M9, T2).

*Photos on fridges, stopped staff from putting things all over the place. Even the posters in the takeaway on how the shelves should look, staff refer to these* (M3, T-mid) .

#### Characteristics of Individuals

The CFIR constructs that were most frequently identified as characteristics of the Store Managers that facilitated implementation were knowledge and beliefs, self-efficacy and other personal attributes (Fig. 1).

Knowledge and Beliefs: All Store Managers, except for two managers of stores that implemented the modified 6-point strategy, indicated belief in the strategy’s efficacy and benefits to the community.

Self-efficacy, and Other Personal Attributes: High confidence was reported by Store Managers in their retail skills to maintain the strategy. They described adeptness in modifying routine store operations to accommodate the strategy such as adjusting stock controls and ordering. They provided hands-on training and encouragement to their staff to maintain the strategy and demonstrated pragmatism, optimism, adaptability and resilience, marketing and retail competency, and knowledge of current sales and their customers purchasing patterns. Lack of knowledge of the local language however in some cases was a barrier for Store Managers to adequately explain the strategy to staff. Letting go of old retail habits was also a barrier for some.

*getting out of old habits…as a retailer you always push your top selling items and it’s hard to get away from that* (M6, T2).

#### Process

Engaging, and Customer Response were the constructs most frequently referred to by Store Managers (Fig. 1).

Engaging – Opinion Leaders, and Champions: Eight of the ten Store Managers described their positive engagement with their staff as central to the strategy’s success. This was expressed as “being on top of what staff are doing” (M8, T-mid), to “…walk with them, make sure they follow the strategy” (M6, T-mid), to “make sure the staff understood why” (M4, T2), and “teaching the staff to stick to a layout” (M7, T2). In contrast, one Store Manager who showed enthusiasm for the strategy and in a store with the modified 6-point strategy thought their staff to not “have any idea” (M10, T2) about the strategy. Half of the Store Managers recognised the positive influence of their staff on the strategy’s success. Another Store Manager referred to the positive impact the Store Board had on staff engagement after explaining the strategy to them in their language (M2, T2).

Customer Response: Managers were sensitive to customer feedback and expressed surprise at the lack thereof. Some found it challenging at the start of the strategy to familiarise customers with the change in product lay-out.

*A lot of people are staring at the shelves where their drink is, they go to grab the [drink name], grab the no sugar and the low sugar* (M9, T2).

*They [customers] don’t really like change, they [customers] get used to things being in places. The drinks at [store 2] have been in place for years* (M4, T2).

*They’ll [customers] still buy biscuits but not more than 1 packet. Before they [customers] might get many types* (M5, T2).

*I thought there would be more, more explanation needed, but I don’t know, they’ve [customers] just all gone with the flow* (M2, T2).

The one Store Manager who received negative comments was optimistic about continuation of the strategy after project completion, stating they would “just deal with it, eventually it will run its course” (M3, T2). Two Store Managers received positive feedback on the strategy from customers including one customer who expressed how the strategy had helped them manage their type 2 diabetes.

*…Another story – a lady came up to [assistant store manager name] and thanked her, the way its [the store] being set up it’s easier to manage her diabetes. She told us that sugar levels started at 14 [mmol/L], ended up at 4 [mmol/L] over 2-weeks just by changing her diet* (M6, T2).

Reflecting and Evaluating: Regular phone contact with the research team member (as part of the fortnightly adherence check) was stated by three Store Managers to be helpful: “Your calling, your support, calling in and not letting me go.” (M6, T-mid) (Fig. 1).

## Discussion

This research provides an in-depth analysis of the factors that operated within and external to the Store Managers to influence the implementation of a novel healthy food retail intervention during a 12-week trial of its efficacy. Factors in each of the five CFIR domains were identified to be important, as was their alignment with each other. This demonstrates the complexity of implementation of such healthy food retail interventions. Elucidation of these factors and their multiple levels of influence, and hence their complexity, can help with the much-needed design and development of implementation strategies to support the adoption and sustainment of evidence-informed health-enabling food retail interventions in to practice by the retail sector.

We found there to be alignment between the Outer and Inner CFIR constructs most frequently referred to by the Store Managers as positive to strategy implementation, and the positive individual and intervention characteristics identified. Networks and communication between the Store Managers and other parts of ALPA, the implementation climate of the ALPA organisation (tension for change, compatibility, relative priority, goals and feedback, learning climate) and its readiness for implementation (organisational commitment shown through leadership engagement, available resources and access to knowledge and information) were CFIR Outer and Inner constructs most frequently referred to by the Store Managers as important to implementation. ALPA prioritised the Healthy Stores 2020 strategy, and from this we identified an organisational readiness for its implementation and a positive implementation climate, that then enabled the flow of necessary communication and resources to Store Managers, who then championed the strategy with their staff.

Implementation climate and ALPA’s readiness for implementation most likely stem from the unique strong sense of social purpose that ALPA has shown with their long history of prioritising community nutrition and health goals. The ALPA Board comprises Aboriginal community representatives who bring to the organisation their commitment to advance the social, cultural, economic and health goals of their community. Health improvement is considered a priority by the ALPA board and the community store a setting to achieve this. This priority for health improvement was also echoed by Store Managers.

Middel et al. (2019) [29] also found the sense of community and health values of retailers to be a strong motivator for the implementation of healthy food interventions. They found that an organisation’s appreciation of the community’s health flowed on to their retailers’ and the retailers’ confidence in the intervention, but that conflict between commercial interests and intervention interests could still present as a barrier to healthy food interventions [29]. Houghtaling et al. (2019) [18] also reported that retailers showed a concern for the communities they served and wished to be responsive to community needs, as did Gupta et al. (2022) [24] who recommended that a further potential implementation strategy may be to provide information to retailers on how interventions may create value for consumers whilst maintaining profitability.

In our study, in contrast to that found by Middel et al. (2019) [29], we found there to be compatibility of the Healthy Stores 2020 intervention with the commercial interests of ALPA as viewed by Store Managers, as in most cases they considered the benefit to the community and community need to outweigh the cost of strategy implementation. Further, once adjustments were made by retailers to align to the strategy, they perceived the strategy cost to be minimal. Co-design of the strategy is likely to have contributed to this compatibility, as the co-design process considered the alignment of strategy impact, commercial risk, and community need.

Unique to the Healthy Stores 2020 study is the food retail context of ALPA. ALPA is a large Indigenous multi-store organisation with experience and maturity in health-enabling food retail that is reflected in their organisation policy. We showed that ALPA was able to establish the processes needed within their organisational structures to support the success of the Store Managers in strategy implementation. These processes were evident across the different management levels and sectors of ALPA and included the mobilisation of staff for strategy set-up, the ongoing checking of adherence and support to rectify adherence, the provision of a reference guide to Store Managers on healthy/unhealthy foods, and consistent communication from ALPA leadership to Store Management and staff on the importance of the strategy.

We have evidence that ALPA as a result of the success of the Healthy Stores 2020 strategy and agreement by the relevant Store Boards, adopted the Healthy Stores 2020 strategy post-trial in to their organisation’s Health and Nutrition strategy. The organisational size and structure of ALPA, in addition to its maturity and leadership in its prioritisation of health, were likely key factors that influenced ALPA’s decision to adopt and institutionalise the strategy post-trial. It may be more difficult for single-store settings with less experience in prioritising health to adopt such initiatives. However, Middel et al. (2019) [29] argue that a single-store setting with less structure, can potentially be more flexible and take-on innovation more readily. This was found to be the case by Stead et al. (2020) [27] on evaluation of the implementation of a mandatory standard for limiting unhealthy food products and promotions in hospital settings in Scotland, where although independent shop managers found the standard to be more challenging to implement compared to chain store operators who benefitted from centralised processes for sourcing of new products, planograms, briefing materials and training, centralised processes constrained the agility needed to adapt to individual shop characteristics and contexts [27]. In the case of ALPA however, the centralised structures and support processes, which customise for context, enabled agility for Store Managers to implement the strategy in the unique context of their store and community.

Store Managers were a ‘make-or-break’ touchstone of implementation success. Our research shows how individual characteristics of Store Managers, including belief in the benefit of the intervention for the community, self-efficacy to adapt existing workflows to fit the strategy, and personal attributes of optimism, adaptability and retail competency, were key to the intervention’s success. This includes the positive influence they had on their store staff. This observation of retailers as facilitators, rather than as impediments to implementation due to their lack of knowledge and/or concern of profit loss, has been a less discussed theme in the literature [18, 24, 29]. Greater understanding is therefore needed of the pivotal role of Store Managers in implementation so as to design implementation strategies that will best support them.

This research provides some insights in to this role. Of note is that although Store Managers championed the strategy, they were not the ones who chose whether or not their store would participate in the strategy, and may not necessarily have been on board at the start. Participation was decided by the Store Boards who represent the community or by the ALPA and Island and Cape Boards for the ALPA-owned stores. The inner setting culture of organisational leadership and commitment to the strategy was clear however, and Store Managers were primed for the strategy through the ALPA Nutritionist and/or Area Manager who communicated what would be involved, and through the teams that spent time with the Store Managers to set-up the strategy in their stores. These supportive elements of effective communication and intervention set-up helped to galvanise Store Manager support, engage the staff, and build Store Manager confidence in the strategy. Boelsen-Robinson et al. (2019) [28] also found in an implementation evaluation of a healthy food policy in a health service setting in Australia, that success in implementation hinged on the provision of resources and support through frequent communication by the health service to the retail staff.

Of further importance in galvanising the Store Manager support in the Healthy Stores 2020 study were the co-designed intervention characteristics of low complexity and cost–benefit alignment. Early and visible signs of intervention impact on sales data reinforced the Store Managers’ trust in the strategy as they could see a cost–benefit. Further aiding Store Manager motivation was the lack of backlash from the community that was expected by some Store Managers as the strategy was unknown ground for them. Enthusiasm for the strategy however was not shared by all Store Managers. While not entirely clear, the data suggest two possible reasons for this. First, a misalignment of the strategy with perceived community need, as viewed by one Store Manager; and second, a cost–benefit imbalance as a result of a perceived low strategy impact, as viewed by this same Store Manager and another. These two Store Managers were in stores that implemented the modified 6-point strategy and may therefore not have experienced the same convincing visible impact on sales mix that some Store Managers described with the complete strategy, although the impact on free sugar was the same for the modified strategy and complete strategy (data not presented). Stead et al. (2020) [27] also reported mixed support for the mandated standard in the Scotland hospital food retail setting. Some managers they interviewed expressed strong support whereas others showed initial ambivalence with concerns about negative consequences on profit. These however largely abated once the changes had bedded in [27].

Accountability to the strategy was important to the Store Managers. Store Managers understood that ALPA expected full adherence, they wanted to be checked and needed feedback that they were doing the right thing. However, moments of non-adherence did occur, despite the high level of commitment from Store Managers and the best efforts from ALPA to clearly communicate the strategy. Provision of resources by ALPA to Store Managers to help implement and monitor the strategy helped to minimise non-adherence as did the fortnightly phone-call adherence checks. Similarly, Stead et al. (2020) [27] found the provision of practical assistance, feedback and guidance on compliance to managers to be important to implementation. Such supports need to be considered in the design of implementation strategies for the adoption of health-enabling food retail by the food retail sector.

## Strengths and limitations

This study uses real-world data to advance knowledge on factors that influenced the implementation of the Healthy Stores 2020 strategy from the perspective of Store Managers responsible for the strategy’s day-to-day implementation. Our use of a well-recognised implementation framework, the CFIR, and consideration of all its constructs in data analysis and reporting, provides a comprehensive basis of implementation barriers and enablers within each of the five CFIR domains that others studies can build on to form a common understanding of factors important to implementation of healthy food retail interventions and from this design effective implementation strategies for their adoption. Interview questions were not guided by the CFIR constructs, meaning that multiple aspects of the CFIR were not asked about. No data for example were coded to the constructs of evidence strength and quality (intervention characteristics), structural characteristics, and culture (inner setting), individual stage of change, and individual identification with organisation (characteristics of individual) and planning, and external change agents (process). Using the CFIR to form interview questions may have helped examine these constructs in more detail. Our interview guide however was purposely designed to be of minimum time burden to very busy Store Managers, and to not pre-empt factors perceived to be of influence to implementation effectiveness. Descriptive data on years in the role and years having worked in Aboriginal and/or Torres Strait Islander communities may have been useful to further examine differences in Store Managers’ experiences of implementation.

Store Managers may have felt pressure to report positively on the strategy due to their employment by ALPA. However, interviews were conducted by the research team and we found there to be diversity in Store Managers’ views of the strategy, with some showing scepticism of the intervention at its start, and two who remained unconvinced of its value for their store. A strength of our study to not be underestimated was the insider knowledge and context-specific experience of the research team. Without this, important contextual factors may have been overlooked in the analysis. Whilst this may lead to bias, this was minimised by cross-checking of codes and themes against interview transcripts and summaries by the research team.

In addition to fortnightly adherence data, we captured Store Manager perceptions, at three different time points of strategy implementation to identify factors influencing implementation at different stages. Interviews of ALPA management, Store Boards, store staff, and customers may also have provided valuable data particularly to examine the role of Store Boards on impacting staff attitudes and customer response. The Store Manager experience however provides rich insight in to how these different players influenced the practice and attitudes of the Store Managers who were directly responsible for day-to-day strategy implementation. This research was conducted with a store organisation where Store Managers were supported by their umbrella organisation. Smaller sized single-store operations may experience different barriers to implementation to those identified herein. It is likely however that regardless of business size or business type (i.e., multiple-store vs single-store), structures and processes similar to those identified in this study, need to be considered in the design of implementation strategies for effective adoption of health-enabling food retail by the food retail sector.

We applied strict criteria to our adherence assessment where even one incident of non-compliance for a strategy component identified at one time point during the 12-week intervention and rectified, was marked as non-adherence. Therefore, while it appears that there was modest implementation of strategy components, such as for the ‘no promotional activity on unhealthy products’ and ‘no visible availability of unhealthy products at high traffic areas’ components, most incidences of non-adherence were rectified when identified. Some evidence of non-adherence at set-up was identified. Future studies would benefit from including an adherence check at set-up to rectify non-adherence at this stage.

### Implications for research and practice

We previously demonstrated the effectiveness of the Healthy Stores 2020 intervention and its resultant impact on reducing sales of free sugars [33]. The co-design approach to this research including the help with set-up, resulted in an intervention and accompanying processes that had a strong fit to the study food retail context and supported its implementation.

There are over 200 food retail stores in very remote Australia and there are factors common to these store contexts, such as community need, that suggest a potential strong fit of the Healthy Stores 2020 intervention. Whilst store structures and internal organisation processes can differ across stores, our research shows that co-design can allow for research evidence to be combined with retailer expertise and organisation structures and processes, to develop a best-fit-to-context intervention and implementation processes to optimise implementation, and one where the cost of implementation is perceived to outweigh the benefit.

Our use of the CFIR provides the food retail research community a unified way to consider and organise the factors that influence implementation of health-enabling food retail initiatives. Further research is warranted to map these factors derived from the CFIR [44] to the long-list of implementation strategies (*n* = 73) for the adoption of evidence-based practice in clinical health care developed by Powel et al. (2015) [45]. This could help build a set of optimal implementation strategies specific to health-enabling food retail. Recent research by Boelsen-Robinson et al. (2021) [46], who applied ‘Systems Thinking’ to interview data collected from four community food retail settings in Australia and identified five implementation stories with 17 associated factors, could also inform this process. Mapping of the implementation strategies to a behavioural change framework such as the Behaviour Change Wheel [47] could then be useful to create theory on what implementation strategies work, why and in what context.

The fortnightly adherence checks were designed primarily to collect data for an assessment of adherence of strategy components. However, they also served to assist with timely rectifying of non-adherence and were an adherence motivator for some Store Managers. This needs to be considered in the design of implementation strategies for effective and wide adoption of health-enabling food retail initiatives. Whilst such a level of monitoring is resource-intensive, frequent monitoring may be required at the start of the implementation of a healthy food retail initiative and then less frequent monitoring and compliance checking continue once a Store Manager is confident with the initiative.

In the future, we will explore the use of video-conferencing for qualitative interviews with Store Managers rather than relying on phone interviews alone. This may be important for research with store businesses where the relationship is early in its establishment.

It is important that there be a workforce specifically trained to support co-design in the food retail context to ensure maximum public health gain from health-enabling initiatives. To address this, we and others are involved in a number of initiatives including: a Centre for Research Excellence in Food Retail Environments for Health (RE-FRESH) [48], an on-line short course for practitioners on transforming food retail environments to be health-enabling [49], a set of co-designed evidence-informed Policy Actions to inform the design of local store policy [50], and a Store Scout App to appraise and provide feedback on store practice against best policy action [51, 52].

The Healthy Stores 2020 intervention that is designed to restrict the promotion of unhealthy food and drinks could benefit communities beyond the food retail context of remote Australia. Large retail companies for example could easily adopt the Healthy Stores 2020 intervention strategy and optimise their existing structures and processes for its effective implementation. The Healthy Stores 2020 strategy however may be more amenable to food retail contexts where there is a strong sense of social purpose, such as that of ALPA, and a close retailer-community relationship, than those where commercial interests primarily drive retail practice.

## Conclusion

This research provides an in-depth analysis of strategy adherence and the factors operating within and external to the Store Managers of the remote community stores that influenced the implementation of the Healthy Stores 2020 strategy. Multiple factors found to operate within each of the CFIR domains, impacted implementation. Overall, positive Store Manager individual and intervention characteristics were underpinned by the unique alignment of the Healthy Stores 2020 strategy with outer community need, the inner sense of social purpose of ALPA and its established organisational structures and processes. Insight in to this complexity of implementation can help optimise the design and development of implementation strategies to support the adoption of food retail interventions in to practice for maximum population health gain. This research provides knowledge on key factors that may be important to implementation strategies for health-enabling food retail interventions in other contexts. ALPA provides an example of how a strong sense of social purpose can reorientate food retail to meet community need.

## Supplementary Information

**Additional file 1: Table A1.** Adherence checklist and photographic material data collection protocol to assess implementation fidelity. **Table A2.** Questions asked to Store Managers by strategy/control stores and time points. **Table A3.** Adherence to Healthy Stores 2020 strategy components, by store.

## Data Availability

The datasets used and/or analysed during the current study are available from the corresponding author on reasonable request.

## Abbreviations

CFIR: Consolidated Framework for Implementation Research
ALPA: The Arnhem Land Progress Aboriginal Corporation
US: United States of America
RCT: Randomised controlled trial
NT: Northern Territory
QLD: Queensland
