# Supplementary material for: Implementation of a food retail intervention to reduce purchase of unhealthy food and beverages in remote Australia: mixed-method evaluation using the consolidated framework for implementation research
Source: Int J Behav Nutr Phys Act. 2023 Feb 17;20:20. doi: 10.1186/s12966-022-01377-y (PMC9938595; doi:10.1186/s12966-022-01377-y)
Supplement: Supplementary file 1 — Additional file 1: Table A1. Adherence checklist and photographic material data collection protocol to assess implementation fidelity. Table A2. Questions asked to Store Managers by strategy/control stores and time points. Table A3. Adherence to Healthy Stores 2020 strategy components, by store. [file 12966_2022_1377_MOESM1_ESM.docx]

**ADDITIONAL FILES**

[Table A1. Adherence checklist and photographic material data collection protocol to assess implementation fidelity 2](#_Toc116021413)

[Table A2. Questions asked to Store Managers by strategy/control stores and time points 4](#_Toc116021414)

[Table A3. Adherence to Healthy Stores 2020 strategy components, by store 6](#_Toc116021415)

## Table A1. Adherence checklist and photographic material data collection protocol to assess implementation fidelity

| **Strategy component** | **Photographic data collected by research team at baseline and follow-up (instructions for collection)** | **Adherence checklist (fortnightly phone calls to store managers & photographs requested every 4 weeks)** |
| --- | --- | --- |
| **1. No promotional activity on RED products** | Photograph any bundle deals (2-for-1 etc), or free product giveaway offers, and promotional discounts or markdowns, instore posters, branding on shop displays such as refrigerators, stickers, wobblers and shelf stripping; external community notices or catalogues. | Questions asked:  Do you have any markdowns or price deals on products at the moment? Think about all areas of the store, including aisle ends, shelves, fridges and freezers, counters and floor stacks. If yes: Which products? How are these labelled? |
| **2. No misleading promotional activity** | Photograph any misleading signage e.g., on posters, fridge branding, shelf strips, stickers. Photograph all healthy and unhealthy drinks facings. | Not asked – some identified through photographs of drinks fridges. |
| **3. No visible availability of RED products at high traffic areas** (Substitute healthier products) | Photograph any potential high traffic areas in the store: Front counters; any areas at the front of store, in between the aisles and the checkout/front of the store, e.g., shelving where customers queue to use checkout; fronts of aisles, including wing stacks; ends of aisles, including wing stacks - only include if backing onto fridges; any free standing ‘floor stacks’ throughout the store; 1m^2^ area surrounding ATMs | Photographs requested:   1. Fronts of aisles including aisle wings/wing stacks 2. Backs of aisles including aisle wings/wing stacks [if high traffic area/facing fridges – check for each store before call] 3. Front counters, and front of the store, in between fronts of aisles and counters 4. ATM (1m area around) if store has ATM |
| **4. Reduced facings table sugar, confectionery & sweet biscuits (**Substitute healthier products) | Photograph facings of table sugar, confectionery and sweet biscuits, ensuring photographs clearly show the number of bays/shelves, including products around the store, e.g. in floor stacks or at front counters. | Photographs requested:   1. Table sugar section 2. Sweet biscuit section   Confectionery section, chocolate section, any other confectionery around the store (note - any other confectionery around the store - no increased facings/space (often no extra photos supplied so difficult to tell). |
| **5. Reduced refrigerator space for targeted drinks (**Substitute healthier drinks) | Photograph all drinks fridges: Takeaway fridges; grab & go fridges near counter/s; free standing fridges; built-in fridges. Number of fridges, shelves per fridge and all products should be clearly visible from photos.  Photograph all healthy and unhealthy drinks facings. Photograph all drinks fridges: Takeaway fridges; grab & go fridges near counter/s; free standing fridges; built-in fridges. Number of fridges, shelves per fridge and all products should be clearly visible from photos. | Photographs requested:  Drinks fridges (take a photo of each 2-door fridge) |
| **6.** **No RED soft drinks >600ml in refrigerators** | Photograph all drinks fridges: Takeaway fridges; grab & go fridges near counter/s; free standing fridges; built-in fridges. Number of fridges, shelves per fridge and all products should be clearly visible from photos. | Photographs requested:  Drinks fridges (take a photo of each fridge)  Questions asked: Where are you keeping the 1.25L sugary drinks? |
| **7. Floor stickers (showing amount of sugar per 1.25L soft drink; promoting water as the healthiest choice) & shelf stripping (giving a warning of high sugar) applied on table sugar, sweet biscuit and confectionery shelves** | (survey visits only): Photograph red ‘sugar in drinks’ floor sticker; green ‘water is the best choice’ floor sticker; ‘warning too much sugar’ shelf stripping in: table sugar section; sweet biscuits shelves; confectionery shelves; chocolates shelves/fridge. | Questions asked: Do you have ‘warning too much sugar’ stripping on all table sugar shelves, confectionery shelves, sweet biscuit shelves?  Are the floor stickers in place: a) sugar in 1.25L drinks b) water is the best choice  Additionally, shelf stripping was checked using the photographs outlined in strategy 4. |

## Table A2. Questions asked to Store Managers by strategy/control stores and time points

| Qualitative Question | Strategy/ control | Time point |
| --- | --- | --- |
| 1. What impact do you see the Healthy Stores 2020 strategies having on food and drink sales? | Strategy | One to three weeks following set-up, mid-trial, and final week of trial |
| 1. What impact do you see the Healthy Stores 2020 strategies having on overall store sales? | Strategy | One to three weeks following set-up, mid-trial, and final week of trial |
| 1. How do you see these strategies helping your store? | Strategy | One to three weeks following set-up, mid-trial, and final week of trial |
| 1. How do you see these strategies helping your customers? | Strategy | One to three weeks following set-up, mid-trial, and final week of trial |
| 1. Have you seen or heard any customer response to the strategies so far? If so, what? | Strategy | One to three weeks following set-up, mid-trial, and final week of trial |
| 1. Which specific strategies or strategy elements are working well so far? | Strategy | One to three weeks following set-up, mid-trial, and final week of trial |
| 1. What has helped you the most with maintaining the strategies to date? (e.g supportive store staff, resources e.g ALPA reference guide?) | Strategy | One to three weeks following set-up, mid-trial, and final week of trial |
| 1. What has been the hardest part of strategy maintenance for yourself as store manager? | Strategy | One to three weeks following set-up, mid-trial, and final week of trial |
| 1. What has been the hardest part of strategy maintenance for store staff? | Strategy | One to three weeks following set-up, mid-trial, and final week of trial |
| 1. [If not answered above] Which specific strategies or strategy elements have not worked well and why? | Strategy | One to three weeks following set-up, mid-trial, and final week of trial |
| 1. What strategies are not being implemented (partially or fully?) and why? | Strategy | One to three weeks following set-up, mid-trial, and final week of trial |
| 1. [If strategies identified above as difficult, refer to these here; also refer to those not mentioned] What do you see as the main barriers to continuing to implement these [specific; and all] strategies for the 12-week period? | Strategy | One to three weeks following set-up, mid-trial |
| 1. Do you foresee that your store can continue any or all of these strategies AFTER the project period? If not, why? | Strategy | Final week of trial |
| 1. Would you have been prepared to also take on the Healthy Stores 2020 strategy that removes 1.25L full-sugar soft drinks from the fridges and place them at ambient temperatures? | Strategy stores with competitor store | Final week of trial |
| 1. Your store has been implementing a range of strategies for the 12-week HS2020 period. What other things do you think could help improve healthy food and drink merchandising in your store? | Strategy | Final week of trial |
| 1. [Other than HS2020] Is there any activity happening in the store and/or community that could influence food and drink sales? (e.g government programs, funerals, sports carnivals, festivals, disruptions to electricity, other population movement, nutritionist activity. ) | Strategy/control | One to three weeks following set-up, mid-trial, and final week of trial |
| 1. I will be calling in a fortnight, please let me know your availability or if you will be on leave, who I will need to contact? | Strategy/control | One to three weeks following set-up, mid-trial, and final week of trial |

## Table A3. Adherence to Healthy Stores 2020 strategy components, by store

|  | **Store Number** | | | | | | | | | | | | | | | | | | | |
| --- | --- | --- | --- | --- | --- | --- | --- | --- | --- | --- | --- | --- | --- | --- | --- | --- | --- | --- | --- | --- |
| **Adherence measure** | **1** | **2** | **3** | **4** | **5** | **6** | **7** | **8** | **9** | **10** | **11** | **12** | **13** | **14** | **15** | **16** | **17** | **18** | **19** | **20** |
| **No promotional activity on RED products**^1,2^ | **0** | **1** | **0** | **0** | **1** | **0** | **1** | **1** | **0** | **0** | NA | NA | NA | NA | NA | NA | NA | NA | NA | NA |
| *Meeting strategy requirements at T1* | 🗸 | 🗸 | x | x | x | x | 🗸 | x | x | x | 🗸 | x | x | x | x | 🗸 | x | x | 🗸 | x |
| *Adherence during T2-T3* | x^3^ | 🗸 | x^4^ | x^5^ | 🗸 | x^6^ | 🗸 | 🗸^7^ | x^8^ | x^9^ | x | x | x | x | 🗸 | x | x | x | 🗸 | x |
| *No promotional shelf stripping on RED products in drink fridges – meeting strateg requirements at T1*^10^ | x | x | x | x | 🗸 | x | x | x | x | x | x | x | x | x | x | x | x | x | x | x |
| *As above – adherence during T2-T3* | x | x | 🗸 | x | 🗸 | x | x | x | x | x | x | x | x | x | x | x | x | x | x | x |
| **No misleading promotional activity**^11^ | **0** | **1** | **0** | **1** | **0** | **1** | **0** | **1** | **1** | **1** | NA | NA | NA | NA | NA | NA | NA | NA | NA | NA |
| *Meeting strategy requirements at T1* | x | x | x | x | x | x | x | x | x | x | NA | NA | NA | NA | NA | NA | NA | NA | NA | NA |
| *Adherence during T2-T3* | x | 🗸 | x | 🗸 | x | 🗸 | x | 🗸 | 🗸 | 🗸 | x | x | x | x | x | x | x | x | x | x |
| **No visible availability of RED products at high traffic areas (Substitute healthier products)^12^** | **0** | **1** | **1** | **0** | **1** | **0** | **1** | **0** | **1** | **1** | NA | NA | NA | NA | NA | NA | NA | NA | NA | NA |
| *Meeting strategy requirements at T1* | x | x | x | x | x | x | x | x | x | x | x | x | x | x | x | x | x | x | x | x |
| *Adherence during T2-T3* | x^13^ | 🗸 | 🗸^14^ | x^15^ | 🗸 | x^16^ | 🗸 | x^17^ | 🗸 | 🗸 | x | x | x | x | x | x | x | x | x | x |
| **Reduced facings^18^ T1-T3 for confectionery and sweet biscuits and table sugar stocked to one bay or less, no multipacks displayed and smaller units at eye level T2-T3** | **1** | **1** | **1** | **1** | **1** | **1** | **0** | **0** | **0** | **1** | NA | NA | NA | NA | NA | NA | NA | NA | NA | NA |
| ***Reduced facings table sugar meeting strategy requirements at T1^19^*** | *x* | *x* | *🗸* | *x* | *🗸* | *🗸* | *x* | *🗸* | *🗸* | *🗸* | *x* | *x* | *x* | *x* | *🗸* | *🗸* | *🗸* | *x* | *🗸*^20^ | *x* |
| ***Reduced facings table sugar compliance during T2-T3*** | *🗸* | *🗸*^21^ | *🗸* | *🗸* | *🗸* | *🗸* | *🗸* | *🗸* | *🗸* | *🗸* | *x* | *x* | *x* | *x* | *🗸* | *🗸* | *🗸* | *x* | *🗸* | *x* |
| ***Confectionery Facings T1*** | *210* | *156* | *104* | *92* | *83* | *107* | *79* | *99* | *52* | *199* | *109* | *70* | *106* | *94* | *47* | *92* | *71* | *44* | *105^22^* | *106* |
| ***Confectionery Facings T2*** | *104* | *88* | *90* | *64* | *75^23^* | *83* | *77^24^* | *76* | *45* | *91* | *NA* | *NA* | *NA* | *NA* | *NA* | *NA* | *NA* | *NA* | *NA* | *NA* |
| ***Confectionery Facings T3*** | *121* | *76* | *64* | *75* | *42* | *81* | *82* | *86* | *37* | *91* | *104* | *70* | *125* | *87* | *65^25^* | *135* | *82* | *48* | *71* | *134* |
| ***Sweet Biscuits Facings T1*** | *35* | *57* | *46* | *25* | *18* | *33* | *18* | *23* | *5* | *49* | *44* | *39* | *20* | *39* | *24* | *29* | *26* | *44* | *18^26^* | *30* |
| ***Sweet Biscuits Facings T2*** | *26^27^* | *37* | *25* | *18* | *12* | *20* | *13* | *24* | *5* | *21* | *NA* | *NA* | *NA* | *NA* | *NA* | *NA* | *NA* | *NA* | *NA* | *NA* |
| ***Sweet Biscuits Facings T3*** | *24* | *31* | *24* | *19* | *13* | *19^28^* | *12* | *25* | *7* | *21* | *43* | *32* | *21* | *44* | *22* | *26* | *27* | *51* | *18* | *33* |
| **Reduced refrigerator facings^29^ for targeted drinks, T1 to T2 and T1 to T3^30^** | **0** | **1** | **1** | **1** | **1** | **1** | **0** | **1** | **0** | **0** | NA | NA | NA | NA | NA | NA | NA | NA | NA | NA |
| ***RED drinks facings T1*** | *56* | *63* | *55* | *60^31^* | *60* | *63* | *58* | *52* | *54* | *62* | *64* | *58* | *57* | *53* | *51* | *60* | *59* | *NA^32^* | *54* | *66* |
| ***RED drinks facings T2*** | *57^33^* | *53* | *47* | *52* | *52* | *52* | *61* | *47* | *52* | *55* | *NA* | *NA* | *NA* | *NA* | *NA* | *NA* | *NA* | *NA* | *NA* | *NA* |
| ***RED drinks facings T3*** | *54* | *51* | *42* | *44* | *43* | *45* | *67^34^* | *45* | *56* | *62* | *61* | *48* | *57* | *53* | *62* | *62* | *62* | *NA* | *55* | *63* |
| **No RED soft drinks >600ml in refrigerators^35^** | **1^36^** | **1** | **1** | **1** | **1** | **1^37^** | **NA** | **NA** | **NA** | **NA** | NA | NA | NA | NA | NA | NA | NA | NA | NA | NA |
| **Floor stickers (showing amount of sugar per 1.25L soft drink; promoting water as the healthiest choice) & shelf stripping (giving a warning of high sugar) applied on table sugar, sweet biscuit and confectionery shelves**^38^ | **1** | **1** | **1** | **0**^39^ | **1** | **1** | **1**^40^ | **1** | **1** | **1** | NA | NA | NA | NA | NA | NA | NA | NA | NA | NA |
| **Total score** | **3** | **7** | **5** | **4** | **6** | **5** | **3** | **4** | **3** | **4** | NA | NA | NA | NA | NA | NA | NA | NA | NA | NA |
| **Score out of** | **7** | **7** | **7** | **7** | **7** | **7** | **6** | **6** | **6** | **6** | NA | NA | NA | NA | NA | NA | NA | NA | NA | NA |
| **Implementation Percent (%)** | **43** | **100** | **71** | **57** | **86** | **71** | **50** | **67** | **50** | **67** | NA | NA | NA | NA | NA | NA | NA | NA | NA | NA |

Key: T1 = baseline. T2 = strategy setup. T3 = end of intervention period. Note that for strategy stores, where ‘Adherence during T2-T3’ is noted, data were collected for strategy stores between T2 & T3; for control stores data is for T3 only. Weeks refer to weeks since setup for intervention stores, and weeks since setup in the first intervention store for control stores. *🗸* = yes; adherence. x = non-adherence at least once at this/these timepoint/s

Stores were coded as 1 for each of the strategy activities if observed to adhere fully to the strategy component at all observed time points (except T1). Data checks on drinks were completed by a second research assistant on six stores for T1, T2 and T3 timepoints (5 strategy stores [2 with competitor stores] and 1 control store) and on confectionery and sweet biscuit data for five stores. Where there was a difference of >3%, both data sets and the accompanying photos were rechecked.

Missing items: Missing products (i.e., shelf space dedicated to a product but stock not available at the time of observation) were estimated or excluded from analysis depending on whether the surrounding shelf space indicated where the product type would normally be situated (through labels, surrounding products, etc).

^1^Including no price discounts, volume promotions (e.g. two for one type offers), posters etc

^2^Infrastructure items exempted from intervention in all stores: branding on ice cream freezers and pie warmers; menu boards in takeaway; branded discretionary products on fridges (note only 3 fridges across all stores).

^3^2 items: week 5 of intervention, promotional price on cartons of assorted biscuits. Asked store manager (manager) to remove, actioned. T3: promotional price on 2L juice - identified in person. Manager moved to back of store and removed promotion.

^4^3 items: week 5: promotional price on sweet biscuits and chips– asked manager to remove, actioned both. End of strategy – promotional price on processed meat. Not actioned.

^5^End of strategy: promotional price on ice creams. Not actioned.

^6^Week 3: promotional price on chips, didn’t inform manager until next call in week 5 – had been sold through.

^7^1 item missed at setup: a discretionary (but not targeted) product at front of store with price promotion. This was identified and removed in week 4 of strategy. Marked as compliant as this was an error in setup.

^8^Week 2: price promotion on chips identified; manager informed in week 4 but had already sold through.

^9^2 items - Week 2: price promotion on chips identified; manager informed in week 4; removed promotion. End of strategy: half price meat pies – no action taken.

^10^This item has been reported separately as a common issue of noncompliance including during the intervention period. 5/10 were 100% compliant with this strategy at T2 – it was not communicated clearly to various stakeholders at strategy setup.

^11^Signage on fridge lightboxes with ‘no sugar’ or water and containing sugary drinks were excluded from this strategy. Fridges were provided by the drinks supplier and therefore required to have some product branding. This potentially misleading branding was exempted in lieu of having fridges promoting discretionary products; also as above, existing infrastructure couldn’t be altered.

^12^Excluding infrastructure (ice cream freezers; pie warmers); excluding discretionary drinks in fridges at front of store approved at T2 (coconut water; flavoured water)

^13^2 items: T3 - 2L juice at aisle end (also noted under strategy 1) - manager moved to back of store. Also T3: floor stack of soft drink cans - asked manager to move; not actioned while research team present. Both identified in person.

^14^Discretionary drinks were within 1m of ATM however this was left in place so as not to move infrastructure or place red drinks in another higher traffic area.

^15^2 items - T3 - floor stack of cordial. Research team identified & informed manager; stack remained. Also T3 - sugary gum at front counter - moved off at request of researchers.

^16^2 items: T3 - 2L juice (red item) at front of store in floor stack. Not actioned. Also T3 - tic tacs on front counter – research team member asked staff to remove. Actioned

^17^Noncompliance noted for 1 item at T3 – clear jar of confectionary on counter.

^18^ Specific criteria for calculating confectionery and sweet biscuit facings:

- same product stacked on top of another (eg a box on top of another box of same product) was counted as 1 facing (this was justified by the box underneath not being accessible to the consumer).
- A ‘dumpbin’ (cardboard display unit) containing only one product type was counted as 1 facing; additional product types were counted as additional facings.
- For multiple hanging products, same product hanging in front of the same product was counted as 1 facing; additional facings were counted for each additional product type.
- Nuts were excluded from confectionery as were all dried fruit except salty plum and dried mango (due to excess added sugar and salt in salty plum and dried mango).

^19^Compliant if table sugar facings reduced to <1 bay, no multipacks displayed, and smaller units at eye level.

^20^Photo from 4 weeks after baseline used; baseline photo not available.

^21^Store 2 was approved at setup to reduce sugar from 4 bays to 2 bays. Compliance at T2-T3 was considered to be <2 bays for this store.

^22^Calculated from T1 & week 1 due to incomplete photos at T1.

^23^Calculated from T2 & week 5 as photos of chocolates missing from T2

^24^Calculated from week 5 as photos of confectionery missing from T2

^25^Chocolates photos substituted in from week 6 for chocolates as photos missing from T3, remaining confectionery from T3.

^26^Calculated from week 6 as photos missing from T1.

^27^Sweet biscuit facings calculated from week 5 as data not available from T1.

^28^Sweet biscuit facings calculated from nearest available timepoint (week 5) as end of strategy visit photos missing.

^29^ Drinks were classified into ‘green/amber’ or ‘red’. Targeted beverages were ‘red’ beverages. Each drink unit visible at the front of the fridge was considered to be 1 ‘facing’. Multipacks of water were counted according to the number of bottles facing the front of the fridge. Duplicate products stacked on top of each other, as was sometimes in the case of juice boxes, were counted as single facings. The number of fridge doors were counted for each store at each time point using the photographic data. If a whole fridge (or entire fridge door) compared to other time points appeared to be missing from the photographic data, data were imputed with data from the next closest timepoint available (including the fortnightly store manager photos where provided).

^30^Some stores had incomplete sets of photographic data; where a store was missing a photo of a fridge, data from the same fridge was substituted in from the next closest photo. Three stores (5,8 and 15) didn’t have any photographs of their milk fridges so these could not be included at any timepoint

^31^A 3-door fridge was missing data and excluded from analysis – as this store was an intervention store at baseline, no similar photos could be substituted (later photos included HS2020 intervention).

^32^Refrigerated beverage data not available for store 18

^33^Data not available at setup. Photographs from 3 weeks later substituted for T1

^34^Used data from week 8/12 for T3– data not available from end of strategy due to broken fridge meaning stock shuffling on day of data collection

^35^ Intervention stores in a community without non-ALPA competitor stores were eligible for this strategy

^36^Week 5, 2 separate items: 1.25L creaming soda identified in fridge through photos, asked duty manager to remove, actioned. Had only been in fridge for a few hours.1.25L tonic water in fridge; identified in-person by ALPA nutritionist, asked manager to remove; actioned. Unclear on timeframe for presence in fridge but likely <1 week, issue with stock it was identified as an issue that arose with arrival of new stock.

^37^T3: 1.25L creaming soda identified in fridge through photos, asked manager to remove, actioned. Had only been in fridge for a few hours.

^38^Shelf stripping ‘warning too much sugar’ on targeted products – confectionery, table sugar, sweet biscuits. Some hang-sell confectionery was unable to accommodate stripping. Floor stickers: 1 indicating quantity of sugar in drinks placed near 1.25L soft drinks; 1 promoting water as the healthiest drink choice placed near water fridge.

^39^This store was missing ‘warning’ stripping on sweet biscuits at setup; this was then set up incorrectly on savoury biscuits instead of sweet. This was identified and manager notified 7 weeks after setup; and actioned 8 ½ weeks after setup

^40^Missing water sticker at setup. Arrived by 2 ½ weeks later (or may have been earlier), therefore compliant
